# The Influence of Norm Perception on Pro-Environmental Behavior: A Comparison between the Moderating Roles of Traditional Media and Social Media

**DOI:** 10.3390/ijerph17197164

**Published:** 2020-09-30

**Authors:** Ruixia Han, Yali Cheng

**Affiliations:** 1 School of Media and Communication, Shanghai Jiao Tong University, 800 Dongchuan RD, Minhang District, Shanghai 200240, China; annahan08@sjtu.edu.cn; 2 Institute of Cultural Innovation and Youth Development, Shanghai Jiao Tong University, 800 Dongchuan RD, Minhang District, Shanghai 200240, China

**Keywords:** norm perception, pro-environmental behavior, traditional media, social media, moderating role, environmental governance

## Abstract

The activation of norm perception can promote pro-environmental behavior. How does media, as important variables in activating norm perception, affect pro-environmental behavior? Through an online survey of 550 randomly selected Chinese citizens, this study examines the roles of traditional media and social media in influencing the relationship between norm perception and pro-environmental behavior. Based on multi-level regression analysis of data, this study found that (1) compared with traditional media, social media play a more significant role in moderating the relationship between norm perception and pro-environmental behavior; (2) the promotion of the perception of injunctive norms by traditional media has a negative relationship with pro-environmental behaviors; (3) the activation of subjective norm perception by social media will promote pro-environmental behaviors. According to this research, in the current media environment, we should carefully release pro-environmental information on social media and encourage relevant discussions, and appropriately reduce environment-relevant injunctive normative information on traditional media. The study also discusses the role of media in regulating norm perception and pro-environmental behavior in different cultural contexts.

## 1. Introduction

The global spread of the COVID-19 has brought issues of health-related risk prevention back to public attention, of which environmental protection is one. According to the data released by the Environmental Performance Index (EPI) in 2020, overall, China ranks 120th among 180 countries. Regarding China’s performance in individual indicators it ranks 96th in health, 96th in air quality, and 156th in ecosystem vitality. This indicates the importance of improving environmental quality in China. China proposed the “Ecological Civilization System Reform” in the 2017 Nineteenth Report. However, the launch and implementation of any policy is inseparable from the cooperation and implementation by individual citizens. How to promote environmental protection on a personal level is very important to the success of various environmental protection movements.

Various studies have explored factors influencing personal pro-environmental behaviors. Gifford and Nilsson [1] summarize 18 major personal and social factors affecting pro-environmental behaviors. Personal factors include knowledge experience, personality and self-construal, felt responsibility, cognitive biases, etc. Social factors include urban–rural differences; religion, cultural and ethnic variations; norms, etc. These variables work together to influence pro-environmental behaviors, and it is important to discover the moderating and mediating mechanisms between these factors. Through a meta-analysis of 57 articles, Bamberg and Möser [2] demonstrate that eight core psycho-social variables influence pro-environmental behaviors. They are perceived behavior control, feelings of guilt, problem awareness, social norms, internal contribution, attitude, ethics, and intention. They believe that pro-environmental behavioral intention interferences with other variables’ effect on pro-environmental behaviors. These researchers prove that there are complex correlations between various influencing factors of pro-environmental behavior. It is very important to understand the relationship between different factors, as some of them play a more important role than others. Then, what are these factors, and what are the mechanisms affecting their role in pro-environmental behavior?

There are four theoretical paradigms to analyze factors influencing pro-environmental behavior: rational behavior theory [3], planned behavior theory [4], normative behavior theory [5], and value-belief-norm Theory (VBN) [6], the last of which is based on the theory of normative activation. Each theory has a different focus. For example, rational behavior theory emphasizes the importance of self-interest and believes that people conduct pro-environmental behaviors to reduce personal health risks. The theory of planned behavior emphasizes the importance of behavioral intention. It believes that behavioral intention is the key variable in affecting pro-environmental behaviors, while the influencing factors of individual behavioral intention may be multifaceted. The theory of normative activation focuses on activating internal norms, such as expectations, stress, and subjective perception. Value-belief-normative theory incorporates the belief in ecological values into the theory of normative activation. Generally speaking, rational behavior theory and planned behavior theory are often integrated, and various theoretical frameworks also attach great importance to the importance of norms. For example, in the theory of planned behavior, “norm” is considered to be an important variable affecting individual behavioral intention. In the normative activation theory, different types of “norm” become the core variables of discussion. Recent scholarship tends to advocate the integration of these theoretical orientations to form integrated models [2,7,8]. ”Norm” occupies an important position in virtually all of these models.

There are various types of norms. According to their source, they can be divided into descriptive norms and injunctive norms [9]. Some scholars have proposed subjective norms. The different influences of each norm on various environmental behaviors have also become an important part of recent research. For example, Park and Smith [10] study the effect of subjective norms, personal descriptive norms and injunctive norms, social descriptive norms and injunctive norms on organ donation intentions and behaviors. However, we tend to neglect the fact that the formation of norms is also affected by external information and environment. Social media play an increasingly significant role as one source of information that ultimately affects pro-environmental behaviors by influencing the formation of norms. For example, based on a survey of 173 households in Hong Kong, Chan [11] finds that mass media can influence residents’ pro-environmental behavior by affecting their subjective norms. The research by Hynes and Wilson [12] indicates that social media can effectively activate people’s social comparison psychology and promote people’s pro-environmental behavior by improving people’s normative cognition. These studies indicate that media environment does affect people’s social normative cognition and pro-environmental behaviors. In mainland China, the development of social media in recent years is very rapid. Social media have infiltrated into the daily lives of ordinary people. Take the most widely used WeChat as an example. According to WeChat’s official report, the number of WeChat users has exceeded 1.1 billion [13]. What is the impact of social media on pro-environmental behavior in comparison to traditional media? What is the difference between them in changing people’s social norms? What is the specific logic of the use of media to influence social norms and then affect the occurrence of pro-environmental behavior?

## 2. Research Theory and Hypothesis

### 2.1. Social Norm Perception and Pro-Environmental Behavior

The theory of social norms derives from Perkins and Berkowitz’s [14] study of adolescent alcohol abuse behavior. They discover that providing alcoholic adolescents with information about the attitudes of peer groups is far more effective than providing information about the negative consequences of alcoholism itself. Therefore, they advocate that intervention should focus more on improving the health awareness and behaviors of the general public. They discover the existence and great influence of social norms and further develop it into an important theoretical path in the study of behavioral and attitude changes [15,16,17,18]. In practice, social normative intervention has also become an important path of behavioral intervention, such as the use of seat belts [19], prevention of drunk driving [20], prevention of sexual assault [21,22,23] and other issues have achieved significant results. Pro-environmental behavior is one of these behaviors.

Pro-environmental behavior refers to environmentally beneficial behaviors that people exhibit in their daily lives, that is, behaviors that tend to be pro-environmental [24]. In general, pro-environmental behavior is influenced by a variety of self-states and external perceptions, such as individual age, gender, knowledge and education, values, politics and worldview, goals, responsibility, childhood experiences, perceived environmental risk perception, environmental knowledge, etc. Normative cognition actually involves the intrinsic specific path of pro-environmental behavior. In this regard, the normative focus theory provides theoretical support. Social psychologists Cialdini et al. [25] suggest that people do a lot of good behaviors not because they have a good sense, attitude or purpose, but because they are constrained by social norms, other people’s behaviors, and typical practices. Taking energy-saving behaviors as an example, they are conducted not so much due to the consideration of environmental protection, social benefit, and money saving than to the influence of the surrounding environment [26]. That is to say, social norms actually affect people’s pro-environmental behavior.

Different social norms affect pro-environmental behavior differently. Social norms are generally divided into two categories, namely descriptive norms and injunctive norms. Descriptive norms refer to the popularity of a certain act, whereas injunctive norms refer to social approval of the act [9]. Reno, Cialdini, and Kallgren [27] argue that injunctive norms can lead people to perform certain behaviors beyond specific socio-cultural contexts and have stronger behavioral guidance. Many scholars have carried out empirical research on different aspects of garbage disposal behavior, energy conservation behavior, conservation and resource conservation behavior, by which they are able to demonstrate the impact of the two norms on various pro-environmental behaviors. However, social norms are not just descriptive norms and injunctive norms. Park and Smith [10] indicate that subjective norms, personal descriptive norms, personal injunctive norms, societal descriptive norms and societal injunctive norms all affect organ donation behavior. They pay special attention to the perceptibility of norms and distinguish the individual from the social aspects. The personal level mainly refers to the influence of family and friends who are in daily contact with people, while the social level mainly refers to the social-cultural environment. Because this study focuses on groups in the same cultural context, it will focus on perceptual descriptive norms and perceptual injunctive norms at the individual level. In addition, Park and Smith [10] also point out that descriptive and injunctive norms are only derived from the division of normative theory. In fact, from the perspective of planned behavior theory, subjective norms should also be included. Subjective norms are designed to capture descriptive norms, i.e., whether important others themselves perform the behavior [28]. We have reason to believe that the norm perception of these three aspects will affect the pro-environmental behavior. Thus, we assume that:

**H1:**  Social norm perception has a positive correlation with pro-environmental behavior;

**H1-1:**  Subjective norm perception has a positive correlation with pro-environmental behavior;

**H1-2:**  Descriptive norm perception has a positive correlation with pro-environmental behavior;

**H1-3:**  Injunctive norm perception has a positive correlation with pro-environmental behavior.

### 2.2. Media Exposure and Pro-Environmental Behavior

The influence of media on pro-environmental behavior has been confirmed by many research institutes. The impact mechanism can be roughly divided into three categories: First, from the perspective of risk communication, media have an amplifying effect on public environmental risk perception, which affects people’s pro-environmental behavior by affecting their attitudes. Agha [29] indicates that the exposure to information about AIDS on mass media amplifies people’s perception of risk and causes them to change behavior. Mileti [30] finds that media communication plays a mediating role between risk perception and pro-environmental behavior. Zeng [31] believes that new media have a greater amplifying effect on environmental risk perception. Understanding of the role of media in affecting people’s risk perception is multi-dimensional. For example, Wahlberg and Sjoberg [32] believe that media do have an impact on risk perception, but the impact is not as heavy as interpersonal communication, and their relationship with behavioral change is not certain. Meanwhile, Fischhoff [33] points out that the relationship between media and risk perception has undergone several stages. In sum, these studies all demonstrate the fact that media influence pro-environmental behavior by affecting people’s risk perception. The second category is from the perspective of use satisfaction theory which points out that media can raise people’s environmental concern, provide environmental-related knowledge, and then affect pro-environmental behavior. For example, Huang [34] discovers that the global warming information obtained by Taiwanese residents from television, newspapers, and the Internet did influence them to behave in a more pro-environmental manner. The works of Holbert et al. [35] and Trivedi, Patel and Acharya [36] both examine the impact of media use on pro-environmental behavior from the perspective of promoting environmental concern. The third category is to analyze the role of media according to the framework of planned behavior theory, subjective norm theory, media dependence and other theories. Among them, Chan [11] combines the influence of mass media on subjective norms and the analysis model of planned behavior theory to analyze the pro-environmental behavior of Hong Kong residents. Lee [37] integrates media exposure in an attitude–intention–behavior model of pro-environmental behavior, and Ho [38] combines planned behavior theory and media dependence theory. Liao et al. [39] test the mediating role of perceived media influence between perceived media exposure of others and perceived social norms. In these studies, the most typical and basic research is the IPMI (the influence of presumed media influence) model [40]. The above research informs us to propose the following hypotheses:

**H2:**  Media usage of environmental information acquisition has a positive correlation with pro-environmental behavior.

By further analyses, we find that the influence of media on pro-environmental behavior shows different characteristics at different stages in the history of media development. For example, in the early days, people mainly focused on the influence of such traditional media types as television, radio, and newspapers on pro-environmental behaviors. Then Internet gradually displayed its influence. In recent years, however, social media have become a new force that initiates their influence on pro-environmental behaviors. If the research of Holbert [35] mainly focuses on TV, the research of Huang [34] compare TV, newspapers and the Internet, while the research of Ho [38] further refers to other media as traditional media to be compared with the Internet. In comparison, the impact of social media on pro-environmental behavior deserves further exploration.

In fact, social media have multiple potentials to influence pro-environmental behavior. For example, Oakley et al. [41], Mankoff et al. [42] have pointed out that the display function of social media reminds the public to follow and promote environmental behavior. At the same time, the recording function of social media also allows the public to have an intuitive feeling about the effects of their pro-environmental behaviors, thereby strengthening their engagement in this aspect of behavior. Social media also function to stimulate social comparison, activate normative perception, and improve pro-environmental behavior [12]. The last advantage of social media is that the information they provide, in comparison to that from official media, attracts more public trust. This helps the construction of online communities and promotes participation in pro-environmental behaviors [43]. Therefore, we argue that social media not only promote pro-environmental behaviors, but also combine the characteristics of media communication with interpersonal communication, and help us perform more than traditional media are capable to do. Therefore, we assume the following:

**H2-1:**  Traditional media have a positive correlation with pro-environmental behavior;

**H2-2:**  Social media have a positive correlation with pro-environmental behavior;

**H2-3:**  The degrees of the influence of social media and traditional media on pro-environmental behavior are different.

### 2.3. Social Norm Perception, Media Composure and Pro-Environmental Behavior

Previous studies have indicated that the perception of social norms can significantly affect pro-environmental behavior. Normative activation theory provides one theoretical support. It is also supported by the focus theory of normative conduct. Relevant behavioral studies have confirmed that regulating people’s norm perception does affect people’s pro-environmental behavior. For example, if the information of the average household electricity consumption in a community is told to those who consume more, they will reduce electricity consumption [44]. This discovery is more significant to the promotion of pro-environmental behavior in countries like China, where it is increasingly difficult to improve people’s environmental awareness and environmental level [45,46]. Under the circumstance when the influencing factors discovered by the planned behavior theory become less influential, it is particularly important to effectively activate public norm perception. The study also finds that perceived descriptive norms and perceptual injunctive norms have different effects on people’s pro-environmental behavior. Taking “discarding garbage” and “zoo feeding” as examples, descriptive norms describe what most people do, whereas injunctive norms look at behaviors’ social consequences in different contexts [27,47]. It is, therefore, particularly important to discover how to arouse people’s normative focus. Subjective norms which develop from planned behaviors are also affecting people’s pro-environmental behavior. Our study pays particular attention to how the three social norms are related to pro-environmental behavior in the Chinese environment.

Social norms can only affect individual behavior when they are perceived and activated. Researchers have explored the connection between social norms and pro-environmental behaviors by examining the mechanism in which social norms are produced and activated. Miller and Prentice [48] conclude that there are three sources of normative beliefs: direct observation of the behavior of others; communication between people or media communication; and speculation of others’ behavior based on personal knowledge. Media, particularly social media, have become more and more important in our lives. Social media combine the functions of traditional mass media with interpersonal communication. While affecting our lives, social media also help adjust our understanding of norms. Existing research demonstrates that media composure does affect people’s perception of social norms and affect people’s pro-environmental behavior. For example, Chan [11] argues that mass media influence pro-environmental behavior by affecting people’s subjective norms. Hynes [12] also indicates that social media indirectly enhance people’s normative cognition by activating people’s social comparative psychology. Whether traditional media and social media activate the perception of social norms in the same way, and how the possible difference affects their influence on pro-environmental behavior are the focus of our research. Therefore, we propose the following:

**H3:**  Media exposure moderates the relationship between norm perception and pro-environmental behavior;

**H3-1:**  Traditional media moderate the relationship between norm perception and pro-environmental behavior;

**H3-2:**  Social media moderate the relationship between norm perception and pro-environmental behavior.

Previous studies demonstrate that different norm perceptions have different effects on pro-environmental behavior. For example, injunctive norm perception is more likely to make people jump out of specific situations and make the norm more effective than descriptive norm perception [27]. This finding is particularly important for the guidance of public behaviors in countries with a low level of pro-environmental behavior. At the same time, we also find that the mechanism of media exposure’s impact on pro-environmental behavior is different. For example, Ho [38] finds that traditional media attention and Internet media attention have different effects on green buying behavior. Chan [11] indicates that television, newspapers and magazines have different effects on subjective norms. The influence and mechanism of social media on norm perception are more likely to be different. For example, it has been found that social media play a role in pro-environmental behavior through social display, social comparison, and public environmental protection efficacy. Because of their own production content, they have a higher influence than the official media. Then, the impact of social media on different norm perceptions may also differ and ultimately affect the environmental behavior, so we assume the following:

**H3-1-1:**  Traditional media moderate the relationship between subjective norm perception and pro-environmental behavior;

**H3-1-2:**  Traditional media moderate the relationship between descriptive norm perception and pro-environmental behavior;

**H3-1-3:**  Traditional media moderate the relationship between injunctive norm perception and pro-environmental behavior;

**H3-2-1:**  Social media moderate the relationship between subjective norm perception and pro-environmental behavior;

**H3-2-2:**  Social media moderate the relationship between descriptive norm perception and pro-environmental behavior;

**H3-2-3:**  Social media moderate the relationship between injunctive norm perception and pro-environmental behavior.

Summarizing these research hypotheses, this study focuses on the following two levels of research questions: RQ1. How do social norm perception and media exposure affect pro-environmental behavior?

RQ2. How does media exposure influence the relationship between social norm perception and pro-environmental behavior? If so, how do social media and traditional media moderate the relationship between different type of norm perceptions and pro-environmental behaviors in the Chinese social context?

## 3. Methods

### 3.1. Data Collection and Sample

This research is mainly based on 550 items of sample data collected through China’s online questionnaire platform Wenjuanxing (Changsha Ranxing Information Technology Co., LTD, Changsha, Hunan province, China). The website’s sample database is composed of 2.6 million people in 30 provincial units in China (Excluding Xinjiang). In this survey, by setting IP addresses, user restrictions, logic questions, etc., and by randomly rolling out questionnaire links, the minimum effective sample number set in this survey is 550. The survey was conducted between August 20 and 27 in 2019. The specific demographic distribution of the survey sample is as follows: 44.7% male and 55.3% female. Taking into account the sex ratio 104.64 (female 100) of China’s actual population, in the subsequent data processing, we carry out a weighted processing. The education distribution is 83.3% for universities and colleges, 11.8% for masters and above, and 2.8% for high schools and below. In terms of income distribution, the annual income of CNY 50,000 to 100,000 accounts for 33.8%, CNY 100,000 to 200,000 accounts for 30.5%, less than CNY 50,000 accounts for 28.1%, more than CNY 200,000 accounts for 7.5%. The average age of the entire sample is 30.5. The specific demographic distribution can be found in our other study [49]. In addition, 83.9% of the samples came from cities, 10.49% from counties and towns, and 5.61% from rural areas.

### 3.2. Measurement

#### 3.2.1. Pro-Environmental Behavior (PEB)

Public pro-environmental behavior is the core variable of this study. There are many measurement methods, such as those of Bratt [50], Gatersleben et al. [51], and Dono et al. [52]. Taking into account the specific social and cultural background of China, we adopt the question series used by Hong et al. (2014) [53] in CGSS (the China General Social Survey) of 2003, 2010, and 2015. This group of questions consists of 10 measurement items, including recycling and discussing environmental issues with relatives and friends [49]. The answers include four items of never, seldom, sometimes and always, to which we assigned 0, 1, 2 3, respectively. The value range of the entire scale is 0–30, and Cronbach’s Alpha is 0.741. The mean is 13.51, and the standard deviation is 3.89. See Table 1.

#### 3.2.2. Social Norm Perception (SNP)

There are various measurement scales for the perception of social norms. This study uses the measurement of subjective norm perception, perceptual descriptive norm, and perceptual injunctive norm in Park and Smith [10]. The research theme has been transformed into specific operations. The specific three categories of measurement problems are as follows: Perception of subjective norms (PSN): (1) my family often does environmental protection things; (2) my friends often do environmental protection things; (3) the general public does a good job in daily environmental protection. The scale internal consistency coefficient Cronbach’s Alpha is 0.715. Perception of descriptive norms (PDN): (1) my family wants me to do things that are good for the environment; (2) my friends want me to do things that are good for the environment: (3) I feel that the general public is engaging in pro-environmental behaviors supervision. Cronbach’s Alpha is 0.740. Perception of injunctive norms (PIN): (1) my family praises what I do for environmental protection; (2) my friends praise me for what is good for the environment; (3) My pro-environmental behaviors have been praised by strangers. The scale’s internal consistency coefficient Cronbach’s Alpha is 0.736. In the data file, we assigned a value of 1–5 according to the frequency of occurrence. Cronbach’s Alpha of the norm perception scale synthesized from three scales is 0.816. See Table 1.

#### 3.2.3. Traditional Media Usage for Environment Information Acquisition (TME) and Social Media Usage for Environment Information Acquisition (SME)

Considering the classification of media types in existing studies [38] and the actual use of media by Chinese residents, the use of traditional media in this study mainly includes magazines, newspapers, television, radio, and the Internet. In particular, based on the history of the Internet, this study separates social media from the Internet as a parallel category. The types of social media usage by Chinese residents in this study mainly include Weibo, WeChat, Tiktok, Kuaishou, Zhihu, QQ, Douban, BaiduTieba, Facebook, Twitter, and Instagram. The specific measurement content includes whether people obtain the four aspects of information through the above media. The specific measurement items refer to our other research [54]. One point is scored for each item. The final score-weighted average value range is 0–4 points. The means of traditional media usage for environment information acquisition (TME) scale is 1.8, the standard deviation is 80, and Cronbach’s Alpha is 0.777. The mean of social media usage for the environment information acquisition (SME) scale is 1.04, the standard deviation is 60, and Cronbach’s Alpha is 0.807. See Table 1.

#### 3.2.4. Other Variables

Other variables in this study include some demographic variables and important variables related with pro-environmental behavior: age, gender, community participation (CP), environmental knowledge (EK), and environmental risk perception (ERP). For operational measurements of gender, age, and community participation, we measured the following: gender: 1 = male, female, and female as control. Age: calculated using 2019 minus the year of birth. Community participation includes: (1) churches, religious groups, (2) sports and fitness groups, (3) cultural and educational groups, (4) professional associations (such as educational associations, business associations), and (5) school-related groups (alumni association), (6) owners’ committee, (7) clan association, family association, association, with 1 point for each participation, ranging from 0–7; the internal consistency coefficient of the scale is 0.754 (Cronbach’s Alpha) [49]. The environmental knowledge level adopts the CGSS2013 version, consisting of 10 measurement items. The internal consistency coefficient is 0.773, and the mean and standard deviation are 9.3 and 1.23, respectively. Environmental risk perception also uses the CGSS2013 version. The internal consistency coefficient of the scale composed of 12 items is 0.818, and the average and standard deviation are 3.74 and 0.93, respectively. For the measurement of the above control variables, please refer to our related research [54]. See Table 1.

### 3.3. Data Analysis Methods and Procedures

This study uses SPSS 19.0 to conduct research hypothesis testing by hierarchical regression. Pro-environment behavior was specifically set as the dependent variable, and then five types of predictive variables were put into the regression model in turn. These five types of predictive variables include (1) demographic variables: gender, age, and community participation; (2) environment-related variables: environmental knowledge and environmental risk perception; (3) norm perception variables, and three types of secondary indicators include: subjective norm perception, descriptive norm perception, and injunctive norm perception; (4) traditional media usage for environment information acquisition and social media usage for environment information acquisition; and (5) interaction variables. We calculated interaction variables by multiplying the relevant variables of norm perception and media exposure.

## 4. Results

### 4.1. Descriptive Statistics

After Pearson’s test of the relevant variables involved in the study, it can be found that for those general variables that affect pro-environmental behaviors, in addition to age (r = 0.152, *p* < 0.01), environmental knowledge (r = −0.116, *p* < 0.01) and environmental risk perception (r = 0.152, *p* < 0.01) are significantly correlated with pro-environmental behavior. Of particular note is that environmental knowledge and pro-environmental behavior show a significant negative correlation. Regarding the correlation between norm perception and pro-environmental behavior, norm perception (r = 0.482, *p* < 0.01) and its three constituent variables, subjective norm perception (PSN) (r = 0.431, *p* < 0.01), descriptive norm perception (PDN) (r = 0.401, *p* < 0.01), and injunctive norm perception (PIN) (r = 0.381, *p* < 0.01), are significantly related to pro-environmental behaviors. In terms of the correlation between media exposure and pro-environmental behaviors, the influence of traditional media exposure (TME) was not significant, while the influence of social media exposure (TME) was significant (r = 0.235, *p* < 0.01). These research results provide an understanding basis for our follow-up regression analysis. It should be noted that because social norm perception is a synthetic variable composed of subject norm perception, descriptive norm perception and injunctive norm perception, its correlation coefficient with these three variables exceeds 0.8 (r > 0.8). See Table 2.

### 4.2. Hypothesis Testing

#### 4.2.1. The Influence of Media Exposure on the Relationship between Norm Perception and Pro-Environmental Behavior

Table 3 shows the results of regression analysis using demographic variables, environment-related variables, norm perception, traditional media usage for environment information acquisition (TME) and social media usage for environment information acquisition (SME), and norm perception and their interaction terms to predict pro-environment behavior. We report the coefficients for the final model and the final R-squared value. From the reported results, it can be found that there is no significant correlation between gender and pro-environmental behavior, that is, there is no significant difference in the performance of pro-environmental behavior between men and women. There is a significant influence of age on pro-environmental behavior (b = 0.073, *p* = 0.000), with older people engaging in more pro-environmental behavior. There is a significant influence of community participation on pro-environmental behavior (b = 0.500, *p* = 0.000), that is, those who are more involved in various types of community organization activities are more engaged in pro-environmental behavior. The model can account for 1.84% of variance in pro-environment behavior.

The relationship between environment-related variables and pro-environmental behavior can be found in M3-2. Both of them have a significant relationship with pro-environmental behavior, but the impact of environmental risk perception on pro-environmental behavior is more significant (b = 1.466, *p* = 0.000).The impact of environmental knowledge on pro-environmental behavior is relatively insignificant (b = −0.288, *p* < 0.05), and in the current sample, environmental knowledge is negatively correlated with pro-environmental behavior.

M3-3 examines the impact of integrating norm perception variables on pro-environmental behaviors. It can be found that norm perception has a significant effect on pro-environmental behaviors, and they are significantly positively correlated (β = 2.498, *p* = 0.000). This result supports H1. The explanatory power of the entire model has been significantly improved, and the adjusted R-squared value has reached 34.6%.

M3-4 examines the impact of traditional media usage for environment information acquisition (TME) and social media usage for environment information acquisition (SME) on pro-environmental behavior. It can be found that the impact of traditional media usage for environment information acquisition (TME) on pro-environmental behavior is near a significant point (b = −0.379, *p* = 0.053), and the impact of social media on pro-environmental behavior is significant (b = 0.875, *p* = 0.001). This partially supports H2 and H2-1, and fully supports H2-2, H2-3. That is, media composure will positively affect pro-environment behavior; traditional media usage for environment information acquisition (TME) has a near-significant impact on pro-environment behavior, but it is negatively correlated; social media usage for environment information acquisition (SME) has a significant positive correlation with pro-environment behavior. The impact of social media on pro-environmental behavior is greater than that of traditional media. 

M3-5 examines the impact of norm perception and media exposure on pro-environmental behaviors. It is found that the interaction effects of norm perception and traditional media usage for environment information acquisition (TME) have no significant impact on pro-environmental behaviors (b = −0.358, *p* = 0.237), but the interaction effect of norm perception and social media usage for environment information acquisition (SME) has a close and significant positive correlation to pro-environmental behavior (b = 0.783, *p* = 0.055), which partially supports H3. H3-1 is not verified, while H3-2 is.

In order to further test the robustness of the results obtained by the above causal step regression method, we use the Bootstrap method to test the main relationship with the help of the process plug-in in SPSS. The results show that without controlling other variables, the mediating effect of social media on norm perception and pro-environmental behavior is significant (R = 0.49, *p* = 0.00), and the LLCI and ULCI are. 0053 and. 0386, respectively. This interval does not contain 0, which suggests that the result is robust. The mediating effect of traditional media on norm perception and pro-environmental behavior is also significant (R = 0.48, *p* = 0.00), but the result is not robust, with LLCI and ULCI of −0.0146 and. 0064, respectively. With the Pearson test results of the pairwise correlation in Table 2, this result suggests the rationality of the strong moderation effect of social media and the possibility of the marginal moderation effect of traditional media without controlling other influencing variables.

#### 4.2.2. The Influence of Media Exposure on the Relationship between Different Types of Norm Perception and Pro-Environmental Behavior

In order to examine in more detail the moderating effect of media environmental information exposure on the relationship between different types of norm perception and pro-environmental behaviors, we conduct further regression analysis to form Table 4. M4-1 and M 4-2 mainly examine the relationship between demographic variables and environment-related variables and pro-environmental behaviors, and the results are consistent with M3-1 and M3-2. M 4-3 examines the relationship between three types of norm perception and pro-environmental behavior. The results show the following: subjective norm perception and pro-environmental behavior are significantly positively correlated (b = 1.197, *p* = 0.000); descriptive norm perception and pro-environmental behavior are significantly positively correlated (b = 0.744, *p* = 0.003); injunctive norm perception was significantly positive correlation with pro-environmental behavior (b = 0.586, *p* = 0.014). This result supports H1-1, H1-2, and H1-3. M4-4 examines traditional media usage for environment information acquisition (TME) and social media environmental information exposure on pro-environmental behaviors. The results show that traditional media have no significant effect on pro-environmental behaviors (b = 0.349, *p* = 0.076), and social media have a significant positive impact on pro-environmental behaviors b = 0.846, *p* = 0.002), once again negating H2-1, supporting H2-2 and H2-3. The results of M 4–5 indicate that the interaction between injunctive norm perception and traditional media environmental knowledge acquisition is significantly negatively correlated with pro-environmental behavior (b = −0.735, *p* = 0.027). The interaction effects (b = −0.040, *p* = 0.903) between subjective norm perception and traditional media (TME) and the interaction effects (b = 0.467, *p* = 0.217) between descriptive norm perception and traditional media (TME) have no significant correlation with pro-environment behavior. The interaction effect (b = 0.964, *p* = 0.048) between subjective norm perception and social media usage for environment information acquisition (SME) is significantly positively correlated with pro-environmental behavior, and the interaction effect (b = −0.168, *p* = 0.757) between descriptive norm perception and social media usage for environment information acquisition (SME) and the interaction (b = −0.192, *p* = 0.676) between injunctive norm perception and social media usage for environment information acquisition (SME) have no significant correlation with pro-environmental behavior. This result supports H3-1-3 and H3-2-1. H3-1-1, H3-1-2, H3-2-2, H3-2-3 are not verified. This means that traditional media play a moderating role between injunctive norm perception and pro-environment behavior, and social media play a moderating role between subjective norm perception and pro-environmental behavior. In other types of norm perception and pro-environmental behavior, neither traditional media nor social media have significant influence. This result more clearly shows the specific mode of traditional media usage for environment information acquisition (TME) and social media usage for environment information acquisition (SME) influencing pro-environmental behavior.

### 4.3. A Summary of the Testing Results of Our Hypotheses

This article aims to examine how norm perception and media composure influence pro-environmental behavior, and whether media composure plays a mediating role between norm perception and pro-environmental behavior. In order to better respond to research questions and report research results more clearly, we have summarized the hypothesis test results, as is shown in Table 5. The table demonstrates that: (1) Norm perception does affect pro-environmental behavior. Different types of norm perception have different effects on pro-environmental behavior. (2) The influence of media exposure on pro-environmental behavior has been confirmed again, although the effect of traditional media deserves further exploration. (3) The influence of media exposure on the relationship between norm perception and pro-environmental behavior is characterized by differentiation. Specifically, traditional media play a negative role in moderating the relationship between injunctive norm perception and pro-environmental behavior, while social media have a positive influence in moderating the relationship between subjective norm perception and pro-environmental behavior.

## 5. Conclusions

This study mainly discusses the role of traditional media and social media in activating public norm perception and influencing pro-environmental behavior in the current media society. The research results show that traditional media usage for environment information acquisition (TME) activates norm perception and affects pro-environmental behaviors less than social media usage for environment information acquisition (SME). Further detailed research discovers that the deepening of public perception of the injunctive norms through traditional media actually reduces people’s pro-environmental behaviors, whereas social media are mainly helpful to activate people’s subjective norm perceptions, thereby promoting pro-environmental behaviors. The influence of environmental knowledge acquisition through traditional media on subjective norm perception and descriptive norm perception has no significant correlation with pro-environmental behavior, and the influence of social media usage for environment information acquisition (SME) on descriptive norm perception and injunctive norm perception has no significant correlation with pro-environmental behavior. The results of this study are very instructive for the promotion of pro-environmental behavior in the age of social media.

### 5.1. Theoretical Implications

#### 5.1.1. In the Age of Social Media, the Role of Different Media Types in the Formation of Pro-Environmental Behaviors Should Be Re-Evaluated

The influence of media on pro-environmental behavior has been confirmed by many studies, and its influence mechanism has also been confirmed by many studies. As mentioned in the literature review, media promote pro-environmental behaviors by amplifying environmental risks [29,31], by increasing people’s environmental concerns and environmental knowledge [34,35,36], and by enhancing people’s value orientation or activating their norm perceptions [11]. In short, media can indirectly affect people’s pro-environmental behaviors in various ways. Relevant research has also found a basis in risk perception theory, use satisfaction theory, acculturation theory, planned behavior and normative activation theory, and has been centrally presented in media dependency theory. However, existing studies have mainly focused on the impact of traditional media, such as television, radio, newspapers, and the Internet on pro-environmental behaviors. Research about the impact of social media on pro-environmental behavior also focuses on the mining of influence mechanisms. For example, social media bring normative pressure through their own social comparison function [12], while improving self-efficacy to promote pro-environmental behavior [41,42]. However, there is no direct research focus on the influence of social media and traditional media on pro-environmental behavior. Our research indicates that social media play a more significant role than traditional media in influencing pro-environmental behavior. In the current era of the media environment, the influence of social media on pro-environmental behavior has become the main way to exert media influence, while the influence of traditional media on pro-environmental behavior is declining.

#### 5.1.2. Interpersonal Communication Holds the Most Significant Effect on Pro-Environmental Behaviors, and Social Media Strengthen This Effect

The influence of interpersonal communication on pro-environmental behavior has long been the focus of many studies. Ho compares respective roles of traditional media and interpersonal communication in media dependence and green purchasing behavior [38]. With many other studies, Ho proves that interpersonal communication plays a greater role than traditional media. Nevertheless, few studies have compared between interpersonal communication and social media. Social media blend characteristics of both interpersonal and mass communication. By interpreting interpersonal communication as interpersonal demonstration effect, our research indicates that social media influence pro-environmental behavior by strengthening interpersonal communication’s demonstration effect. Social media affect pro-environmental behaviors by displaying them publicly. Oakley et al. [41] and Mankoff et al. [42] indicate that social media put small behaviors in daily life under public scrutiny and encourage people’s pro-environmental behaviors by improving public understanding of their own behavior and the behavior of others. Social media also affect pro-environmental behaviors with the recording function, which helps form public awareness of their deeds and efficiency. Social media also affect pro-environmental behaviors by stimulating people’s psychology of social comparison and improving their recognition of social norms [12]. Han et al. [43] points out that user-generated content (UGC) is more likely to gain public trust than official information, by activating pro-environmental norms, creating environmentally friendly online communities, and increasing public pro-environmental participation. Compared to studies which focus exclusively on social media, this study makes a comparative analysis of the mechanism by which interpersonal communication and social media influence pro-environmental behaviors.

### 5.2. Practical Implications

#### 5.2.1. Compared with the Dissemination of Pro-Environment Knowledge through Traditional Media, the Dissemination of Pro-Environment Knowledge through Social Media Has a Greater Effect in Promoting Pro-Environmental Behavior

Our research shows that in the current era of social media, the influence of traditional media on pro-environmental behavior is weakening, while the influence of social media on pro-environmental behavior is increasing. Taking normative perceptual activation as an example, the influence of traditional media on pro-environmental behavior is far less significant than that of social media. At the same time, in the specific types of normative activations, the activation of injunctive norms by traditional media negatively affects pro-environment behavior. Excessively diffusing pro-environment information on traditional media may make people stressful and reduce pro-environmental behavior, which is very close to the negative correlation between the increase in environmental knowledge and the pro-environmental behavior found in this sample. In comparison, disseminating pro-environmental information on social media is helpful to improve people’s supervisory cognition and promote pro-environmental behaviors. In other words, we should pay attention to the diffusion of pro-environmental information on social media, guide people to form discussions on pro-environmental behaviors in social media, and promote public pro-environment behaviors by stimulating social comparison, norm perception and self-efficacy. We should shift the focus of media information promotion for pro-environmental behavior from traditional media to social media.

#### 5.2.2. Reducing the Release of Injunctive Normative Information for Pro-Environmental Behavior in Traditional Media and Increasing Pro-Environmental Information for Promoting Subjective Norm Activation in Social Media Can Promote Pro-Environmental Behavior

This study indicates that there is a complex correlation between normative perceptual activation and media usage. Understanding this relationship is a prerequisite to effectively promote pro-environmental behavior. The research results show that the dissemination of the prescriptive normative information must be appropriately reduced in traditional media. This result is very similar to the effect of excessive “persuasion” in communication research, especially in countries with a strong collectivist cultural tradition. An overemphasis on prescriptive normative information, or the evaluation of others, is likely to cause people’s rebellious psychology and reduce people’s pro-environmental behavior. The activation effect of social media on subjective norms proves that people’s sense of freedom on social media can stimulate their sense of self-efficacy and subjective norms, thereby promoting pro-environmental behavior. This demonstrates the flexible effects of social media on personal and social integration. In the current situation where the relationship between media and people is more and more interactive, paying attention to the activation of people’s subjective norms by social media will be an effective way to improve pro-environmental behavior.

### 5.3. Limitations

The focus of this study is the moderating effect of traditional media and social media on norm perception and pro-environmental behaviors. Therefore, we focus on demographic variables and environmental protection related variables that have significant relationships. There are many factors affecting pro-environmental behaviors. Gifford and Nilsson [1] have summarized the 18 major personal and social factors of pro-environmental behavior, but in order to study the core themes, we select only the variables that are considered most important to this study. Regarding the conceptual configuration of norm perception, we divide it into subjective norm perception, descriptive norm perception, and injunctive norm perception. We mainly draw on the research division proposed by Park and Smith [28]. There are other more reliable and better ways of operation, which are worthy of further research. This study examines the relationship between media’s activation of norm perception and its impact on pro-environmental behavior. In fact, the type of media contact, whether through traditional media or social media, is composed of multiple specific types of media. What are the differences in the activation of specific media types such as WeChat and Facebook? This will be very meaningful for studying the influence mechanism of specific social media in different cultural backgrounds, and it is worth further exploration in future research.

In terms of research methods, this research uses traditional multi-level regression to compare different models, even though there are many types of media and norm perception. If the structural equation method is adopted, the complexity can be more clearly shown. In addition, this study is based on specific practical considerations, and some assumptions that have a significance level slightly higher than 0.05 are marked as significant, which requires special explanation. This study is based on a Chinese sample. Will the role of media contact between norm activation and pro-environmental behavior be different in different cultural contexts? If we find that the activation of injunctive norms by traditional media reduces pro-environmental behaviors, and the activation of subjective norms by social media is conducive to pro-environmental behaviors, then in non-collectivist cultures, will the activation of injunctive norms by traditional media be positive? The influence on pro-environmental behavior needs to be tested by comparative studies.

To conclude, this study indicates that in the current media society, social media play a more important role than traditional media in regulating and promoting pro-environmental behavior. Understanding this role of social media is of great significance for regulating people’s cognition and behavior in an era of constantly changing media environments. The promotion of pro-environmental behavior must rely on the media environment.

## Figures and Tables

**Table 1:** Source and Statistics of main variables.

	Items	Range	Mean	SD	Cronbach α	Source
Pro-environmental behavior	10	0–30	13.51	3.89	0.741	Hong et al. (2014)
Social norm perception	3	0–5	3.51	0.60	0.816	Park and Smith (2007)
Subject norm perception	3	0–5	3.33	0.68	0.715	
Descriptive norm perception	3	0–5	3.53	0.73	0.740	
Injunctive norm perception	3	0–5	3.68	0.73	0.736	
Traditional media exposure	5	0–4	1.8	0.8	0.777	Self-development scale
Social media exposure	11	0–4	1.04	0.6	0.807	
Environmental knowledge	10	0–10	9.3	1.23	0.773	the China General Social Survey 2013
Environmental risk perception	12	0–5	3.74	0.53	0.818	the China General Social Survey 2013

**Table 2:** Pearson intercorrelation of main variables (n = 550).

	1	2	3	4	5	6	7	8	9
Age									
Environmental knowledge	−0.122 **								
Environmental risk perception	−0.070	0.223 **							
Social norm perception	0.028	−0.016	0.211 **						
Subject norm perception	0.010	−0.099	−0.170	0.819 **					
Descriptive norm perception	0.032	−0.011	0.192 **	0.866 **	0.163 **				
Injunctive norm perception	0.029	0.064	0.168 **	0.829 **	0.228 **	0.491 **			
Traditional media exposure	0.010	0.133 **	0.115 **	0.203 **	0.115 **	0.163 **	0.229 **		
Social media exposure	−0.075	−0.011	0.053	0.261 **	0.236 **	0.194 **	0.228 **	0.498 **	
Pro-environmental behavior	0.152 **	−0.116 **	0.152 **	0.482 **	0.431 **	0.401 **	0.381 **	0.070	0.235 **

Notes: ** *p* < 0.01.

**Table 3:** Multiple regression analysis of the influence of norm perception on pro-environmental behavior.

		M3-1	M3-2	M3-3	M3-4	M3-5
	Gender	−0.208 (0.309)	−0.072 (0.303)	−0.044 (0.277)	−0.092 (0.275)	−0.065 (0.275)
	Age	0.073 **** (0.020)	0.058 ** (0.020)	0.058 *** (0.018)	0.066 **** (0.018)	0.063 *** (0.018)
	Community participation	0.500 **** (0.048)	0.497 **** (0.048)	0.364 **** (0.045)	0.342 **** (0.046)	0.347 **** (0.046)
	Environmental knowledge		−0.288 * (0.125)	−0.245 * (0.114)	−0.210 (0.114)	−0.207 (0.114)
	Environmental risk perception		1.466 **** (0.288)	0.816 ** (0.271)	0.818 ** (0.269)	0.785 ** (0.269)
*Independent variable*	Social norm perception			2.498 **** (0.239)	2.399 **** (0.243)	2.263 **** (0.516)
*Moderating variables*	Traditional media exposure				−0.379 * (0.195)	0.902 (1.086)
	Social media exposure				0.875 *** (0.265)	−1.996 (1.516)
*Interaction variables*	Social norm perception × Traditional media exposure					−0.358 (0.302)
	Social norm perception × Social media exposure					0.783 * (0.406)
	F	41.016 ****	31.184 ****	49.391 ****	39.042 ****	31.709 ****
	Adjusted R^2^	0.179	0.216	0.346	0.357	0.359
	ΔR^2^	0.184	0.039	0.130	0.013	0.004

Notes (1) * *p* < 0.05, ** *p* < 0.01, *** *p* < 0.001, **** *p* = 0.000. (2) Unstandardized coefficients are reported (b).

**Table 4:** Multiple regression analysis of the influence of different types of norm perception on pro-environmental behavior.

		M4-1	M4-2	M4-3	M4-4	M4-5
	Gender	−0.208 (0.309)	−0.072 (0.303)	−0.036 (0.277)	−0.084 (0.275)	−0.046 (0.274)
	Age	0.073 **** (0.020)	0.058 ** (0.020)	0.060 *** (0.018)	0.067 **** (0.018)	0.061 *** (0.018)
	CP	0.500 **** (0.048)	0.497 **** (0.048)	0.368 **** (0.046)	0.347 **** (0.046)	0.348 **** (0.046)
	EK		−0.2881 * (0.125)	−0.213 (0.116)	−0.185 (0.116)	−0.185 (0.116)
	ERP		1.466 **** (0.288)	0.800 ** (0.271)	0.803 ** (0.269)	0.776 ** (0.269)
*Independent variables*	PSN			1.197 ** (0.253)	1.093 **** (0.253)	0.217 (0.621)
	PDN			0.744 ** (0.250)	0.746** (0.248)	0.148 (0.643)
	PIN			0.586 * (0.238)	0.581* (0.239)	1.972* (0.566)
*Moderating variables*	TME				−0.349 (0.197)	0.947 (1.068)
	SME				0.846 ** (0.267)	−1.211 (1.534)
*Interaction variables*	PSN × TME					−0.040 (0.329)
	PDN × TME					0.467 (0.377)
	PIN × TME					−0.735 * (0.331)
	PSN × SME					0.964 * (0.487)
	PDN × SME					−0.168 (0.541)
	PIN × SME					−0.192 (0.459)
	F	41.016 ****	31.184 ****	37.421 ****	31.405 ****	20.743 ****
	Adjusted R^2^	0.179	0.216	0.347	0.356	0.365
	ΔR^2^	0.184	0.039	0.133	0.012	0.016

Notes: (1) * *p* < 0.05, ** *p* < 0.01, *** *p* < 0.001, **** *p* = 0.000. (2) Unstandardized coefficients are reported (b).

**Table 5:** A summary of test results.

	Aim	Hypothesis		Model	Result
1	Tests on the relationship between social norm perception and pro-environmental behavior	H1	NP → PEB	M3-3	√
		H1-1	PSN → PEB	M4-3	√
		H1-2	PDN → PEB	M4-3	√
		H1-3	PIN → PEB	M4-3	√
2	Tests on the relationship between media exposure and pro-environmental behavior	H2	ME → PEB	M3-4,M4-4	√
		H2-1	TME → PEB	M3-4	√
		H2-2	SME → PEB	M3-4,M4-4	√
		H2-3	TME, SME → PEB	M3-4,M4-4	√
3	Tests on the moderating effects of different types of information composure on the relationship between different types of norm perception and pro-environmental behavior	H3	NP × ME → PEB	M3-5,M4-5	√
		H3-1	NP × TME → PEB	M3-5	×
		H3-2	NP × SME → PEB	M3-5	√
		H3-1-1	PSN × TME → PEB	M4-5	×
		H3-1-2	PDN × TME → PEB	M4-5	×
		H3-1-3	PIN × TME → PEB	M4-5	√
		H3-1-1	PSN × SME → PEB	M4-5	√
		H3-1-2	PDN × SME → PEB	M4-5	×
		H3-1-3	PIN × SME→ PEB	M4-5	×
